# Supplementary material for: Gas6 Prevents Epithelial-Mesenchymal Transition in Alveolar Epithelial Cells via Production of PGE2, PGD2 and Their Receptors
Source: Cells. 2019 Jun 26;8(7):643. doi: 10.3390/cells8070643 (PMC6678614; doi:10.3390/cells8070643)
Supplement: Supplementary file 1 [file cells-08-00643-s001.pdf]

Online Supplement

Gas6 prevents epithelial-mesenchymal transition in alveolar epithelial cells

Ji-Hae Jung, Ye-Ji Lee, Youn-Hee Choi, Eun-Mi Park, Hee-Sun Kim, and Jihee Lee Kang*

*Correspondence should be addressed to:

jihee@ewha.ac.kr

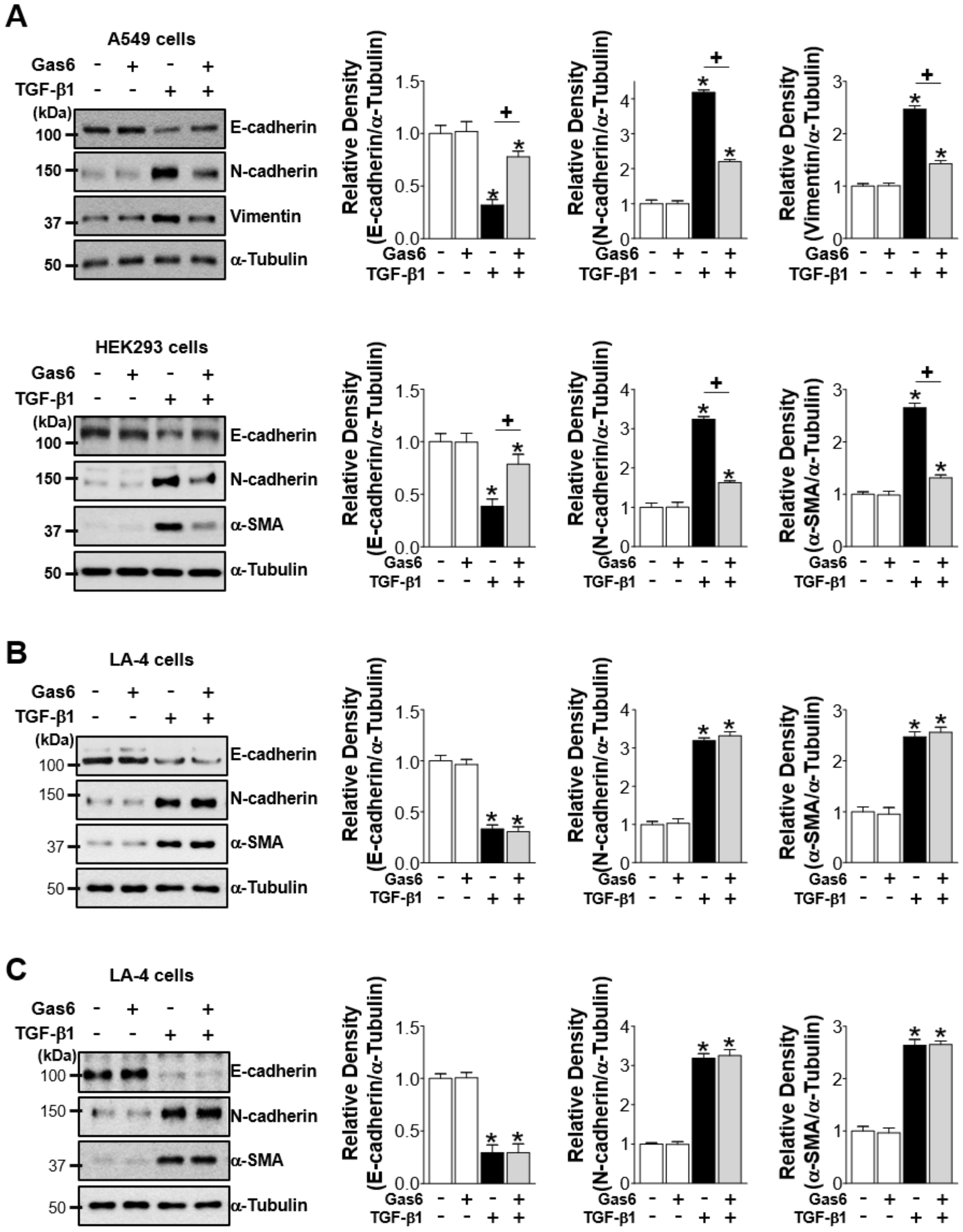

Fig. S1. Effect of Gas6 on TGF-β1-induced epithelial-mesenchymal transition (EMT) in lung and kidney epithelial cells. (A) Immunoblots analysis of the relative amounts of EMT markers in total cell lysates of A549 cells and HEK293 pretreated with 400 ng/ml Gas6 for 20 h and then stimulated with 10 ng/mL TGF-β1 for 72 h. (B) Immunoblot analysis of the relative amounts of EMT markers in LA-4 cells pretreated with 400 ng/ml Gas6 for 2 h and then stimulated with 10 ng/mL TGF-β1 for 72 h. (C)

Immunoblot analysis of the relative amounts of EMT markers in LA-4 cells, which were pretreated with 400 ng/ml Gas6 for 20 h, and then washed out with fresh media before stimulation with 10 ng/ml TGF-β1 for 72 h. Densitometric analysis of the indicated EMT markers' relative abundances. Values represent the mean ± s.e.m. of three independent experiments. $^*P < 0.05$; compared with control; $^+P < 0.05$ as indicated. Results are representative of three independent experiments.

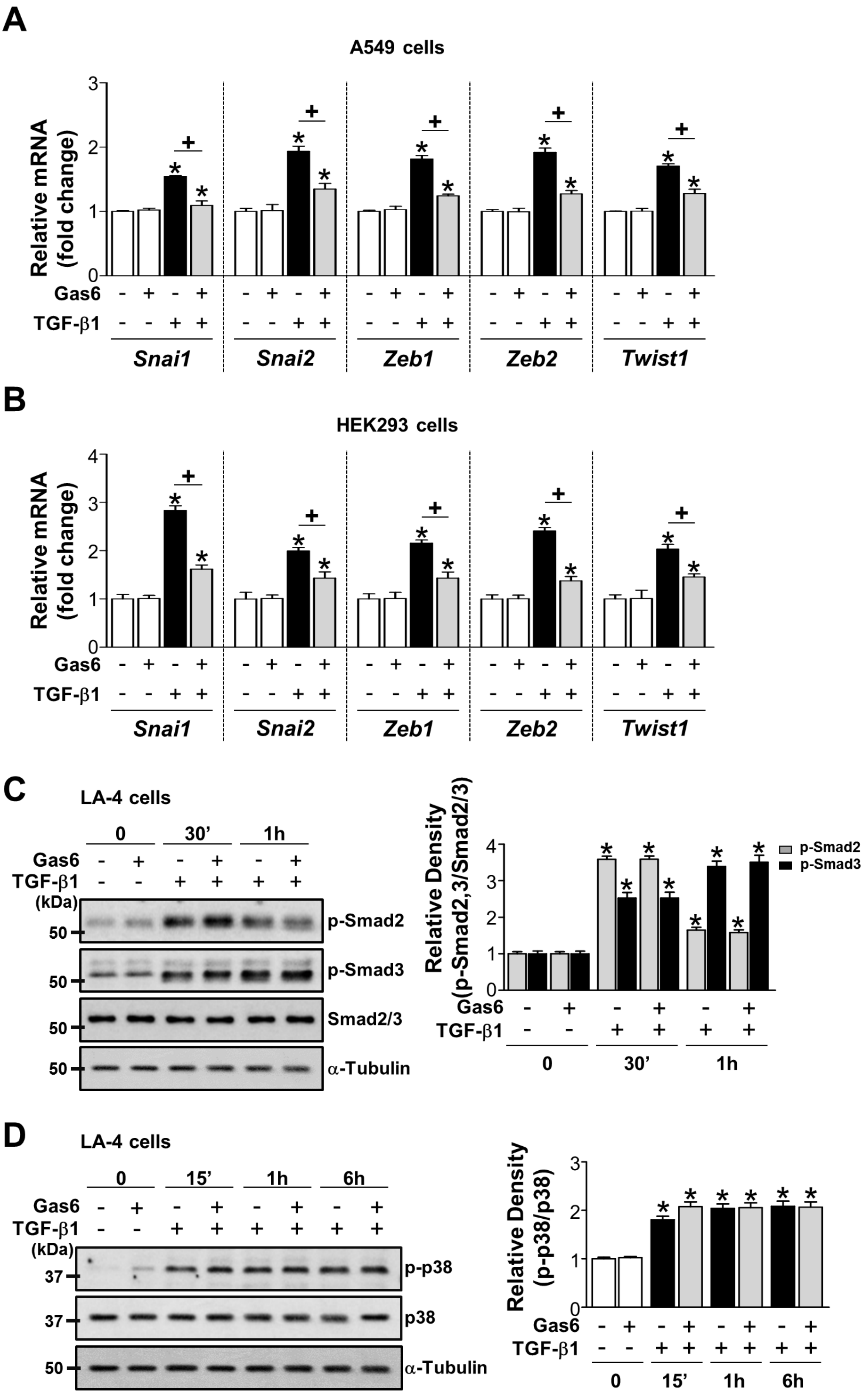
A
A549 cells
Relative mRNA (fold change)
Gas6
TGF-β1
Snai1
Snai2
Zeb1
Zeb2
Twist1
B
HEK293 cells
Relative mRNA (fold change)
Gas6
TGF-β1
Snai1
Snai2
Zeb1
Zeb2
Twist1
C
LA-4 cells
0
30'
1h
Gas6
TGF-β1
(kDa)
50
p-Smad2
p-Smad3
Smad2/3
α-Tubulin
Relative Density (p-Smad2,3/Smad2/3)
p-Smad2
p-Smad3
D
LA-4 cells
0
15'
1h
6h
Gas6
TGF-β1
(kDa)
37
50
p-p38
p38
α-Tubulin
Relative Density (p-p38/p38)

Fig. S2. Effect of Gas6 pretreatment on epithelial-mesenchymal transition (EMT)-regulating transcription factor expression and TGF-β1 signaling in epithelial cells. (A and B) A549 cells and HEK-295 were pretreated with 400 ng/ml Gas6 20 h prior to 10 ng/ml TGF-β1 stimulation for 72 h. qPCR analysis of *Snai1/2, Zeb1/2, and Twist1* mRNAs. (C and D) Immunoblot analysis of the relative amounts of total and phosphorylated Smad2, Smad3, and p38 MAP kinase protein in LA-4 cells over time. Alpha-tubulin was used as a loading control. Densitometric analysis of the relative phosphorylated protein abundances, normalized to that of total protein. Data in all bar graphs are the mean ± s.e.m. of three independent experiments. $^{*}P < 0.05$ compared with control; $^{+}P < 0.05$ as indicated.

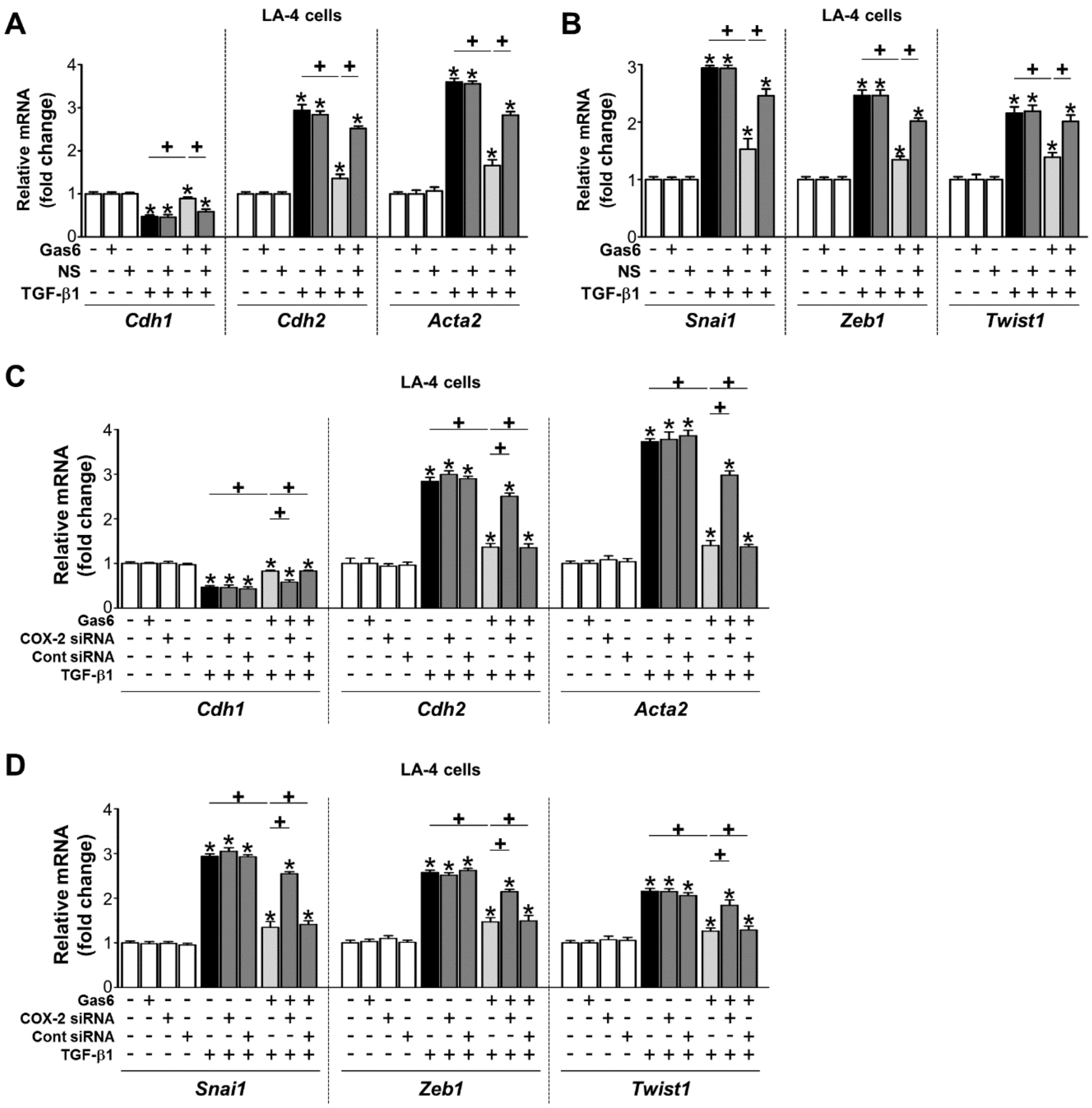

Fig. S3. Cyclooxygenase (COX)-2 signaling is required for Gas6-induced inhibition of epithelial-mesenchymal transition (EMT) in LA-4 epithelial cells. (A and B) LA-4 cells were pretreated with 10 μM NS-398 1 h before 400 ng/ml Gas6 treatment for 20 h and then stimulated with 10 ng/ml TGF-β1 treatment for 72 h. qPCR analysis of EMT markers' and EMT-regulating transcription factors' mRNAs in cell lysates. (C and D) LA-4 cells were transfected with COX-2 specific or control siRNA for 6 h prior to treatment with 400 ng/ml Gas6 for 20 h and then stimulated with 10 ng/ml TGF-β1 for 72 h. qPCR analysis of EMT markers' and EMT-regulating transcription factors' mRNAs in cell lysates. Values represent the mean ± s.e.m. of three independent experiments. **P* < 0.05 compared with control; $^{+}$*P* < 0.05 as indicated.

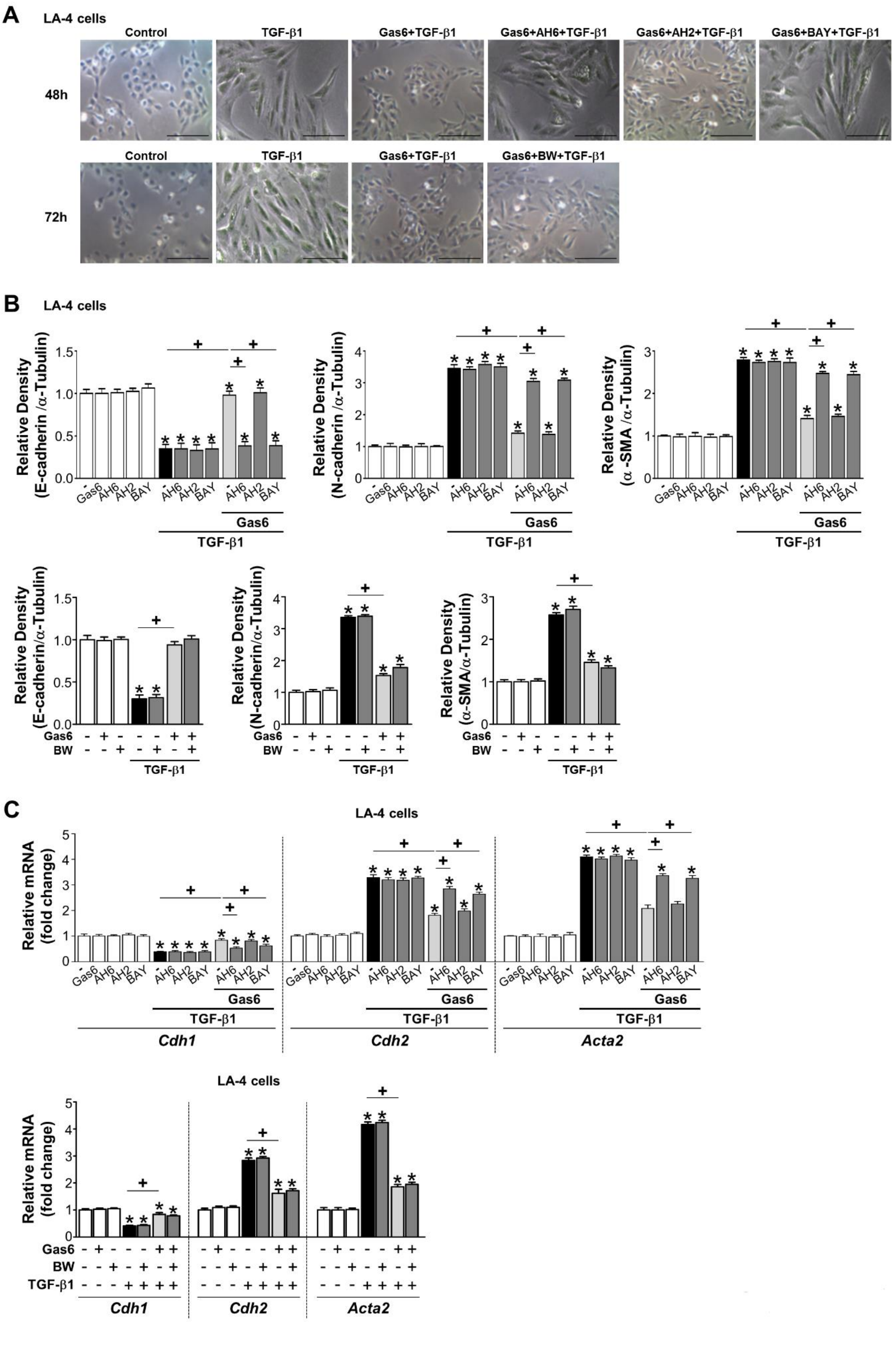
A
LA-4 cells
Control
TGF-β1
Gas6+TGF-β1
Gas6+AH6+TGF-β1
Gas6+AH2+TGF-β1
Gas6+BAY+TGF-β1
48h
Gas6+BW+TGF-β1
72h
B
Relative Density (E-cadherin /α-Tubulin)
Relative Density (N-cadherin /α-Tubulin)
Relative Density (α -SMA /α-Tubulin)
Gas6
AH6
AH2
BAY
TGF-β1
Relative Density (E-cadherin/α-Tubulin)
Relative Density (N-cadherin/α-Tubulin)
Relative Density (α-SMA/α-Tubulin)
BW
C
Relative mRNA (fold change)
Cdh1
Cdh2
Acta2

Fig. S4. Antagonists of prostaglandin (PG)E_2 and PGD_2 receptors reverse Gas6-induced epithelial-mesenchymal transition (EMT) inhibition in LA-4 epithelial cells. (A to C) LA-4 cells were stimulated with 400 ng/ml Gas6 for 20 h and then stimulated with 10 ng/ml TGF-β1 with or without antagonists of EP2 (AH-6809), EP4 (AH-23848), DP1 (BW-A868C), or DP2 (BAY-u3405) at 10 μM. (A) After 48 or 72 h, morphological changes in the cells were examined by phase-contrast microscopy (Scale bars = 50 μm). (B) EMT markers' protein abundances were quantified by immunoblot analysis and normalized to α-tubulin. (C) qPCR analysis of EMT markers' mRNAs in LA-4 cells. Values represent the mean ± s.e.m. of three independent experiments. $^*P < 0.05$ compared with control; $^+P < 0.05$ as indicated.

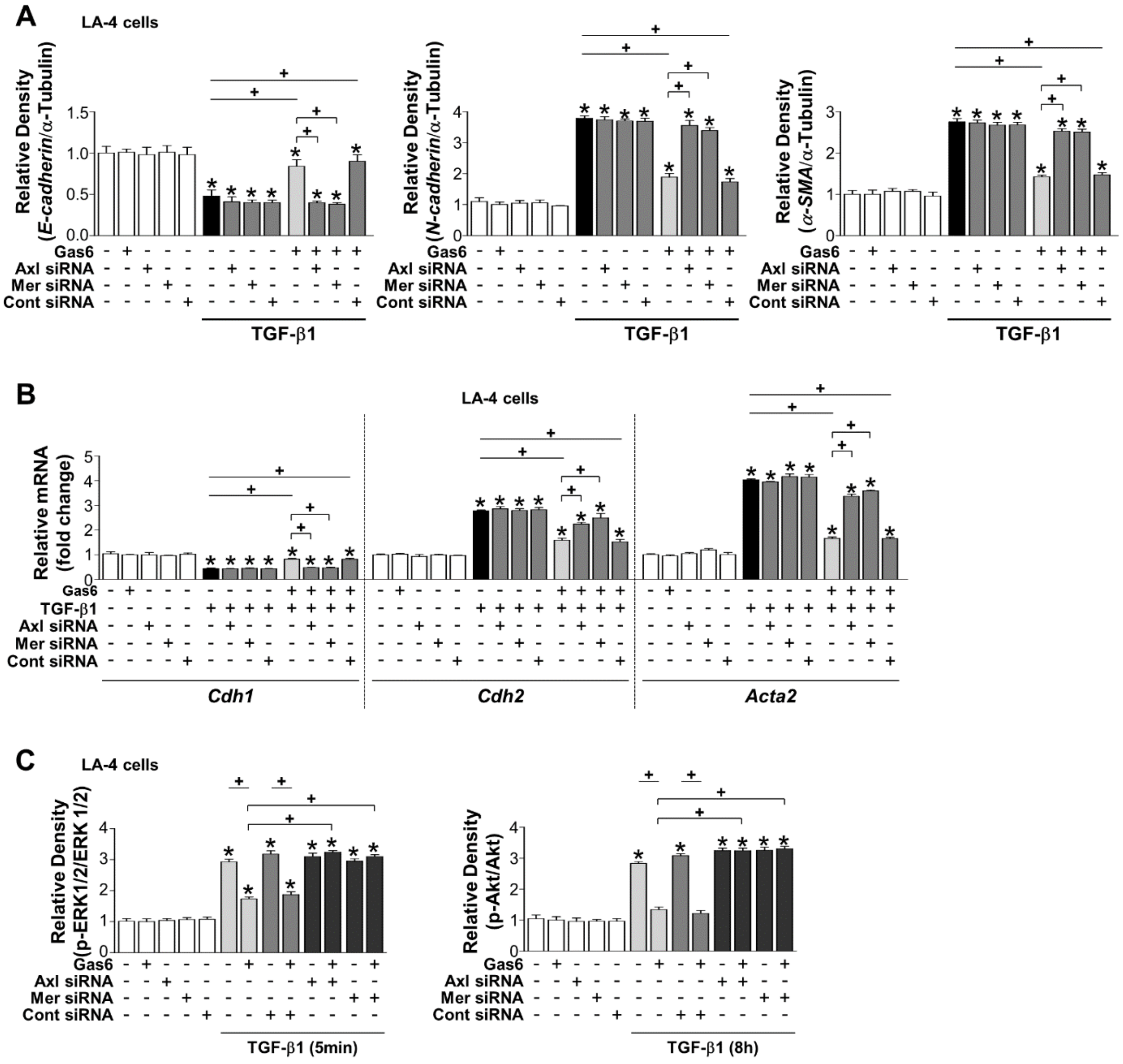

Fig. S5. Inhibition of Axl or Mer signaling reverses suppression of epithelial-mesenchymal transition (EMT) in LA-4 epithelial cells by Gas6. (A to C) LA-4 cells were transfected with Axl, Mer, or control siRNA for 48 h before 400 ng/ml Gas6 treatment for 20 h, and then stimulated with 10 ng/ml TGF-β1 for 72 h or the time indicated. (A) EMT markers' protein abundances were quantified by immunoblot analysis and normalized to α-tubulin. (B) qPCR analysis of EMT markers' mRNAs in LA-4 cells. (C) Phosphorylated ERK and Akt levels were quantified by immunoblot analysis and normalized to total proteins. Values represent the mean ± s.e.m. of three independent experiments. *$P < 0.05$; compared with control; $^{+}P < 0.05$ as indicated.

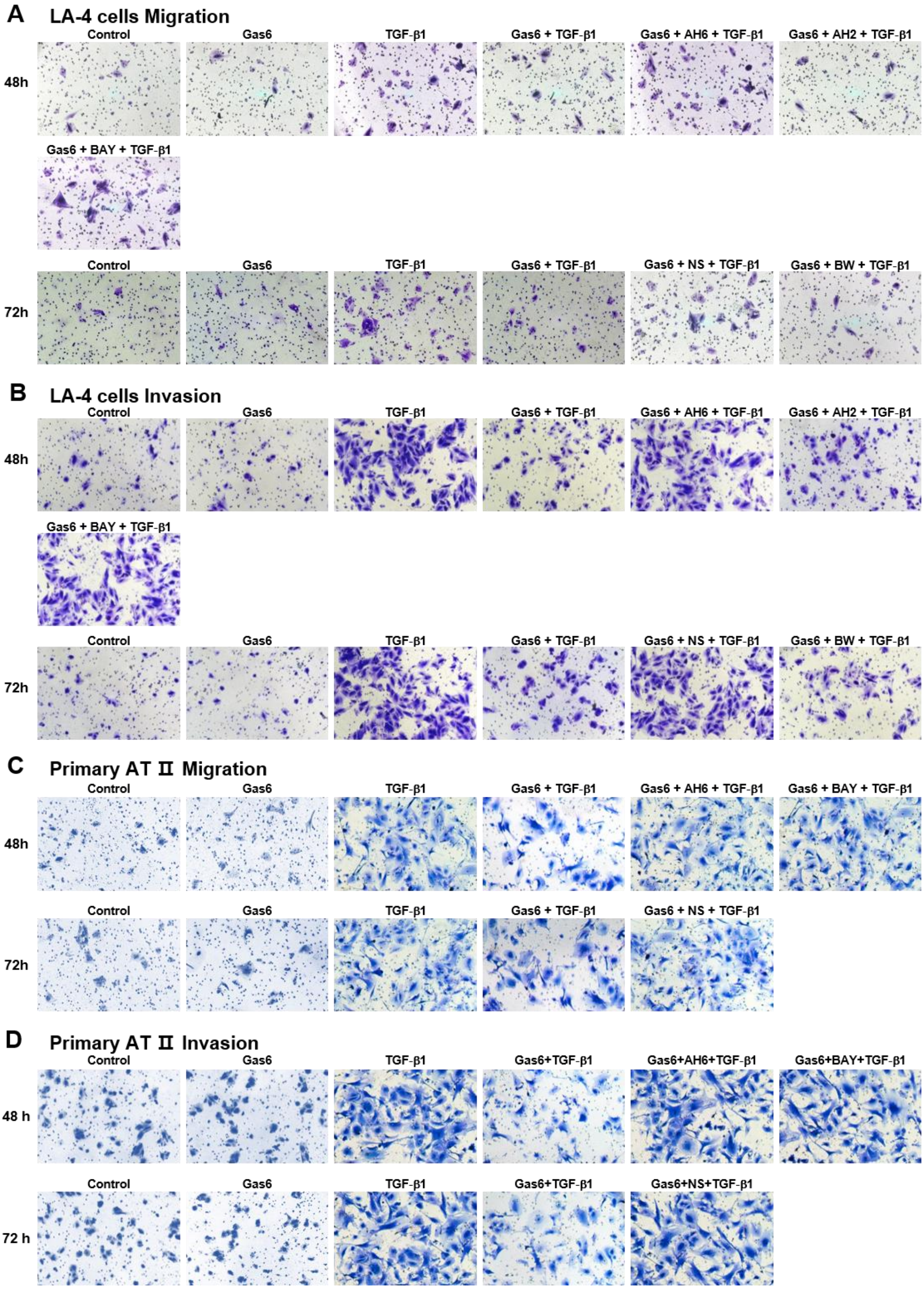

Fig. S6. Gas6 inhibits on TGF-β1-induced migration and invasion through prostaglandin (PG)E$_2$ and PGD$_2$. (A to D) LA-4 and primary alveolar type II epithelial cells were stimulated with 400 ng/ml Gas6 for 20 h in the absence or presence of NS-398 at 10 μM and then stimulated with 10 ng/mL TGF-β1 with or

without antagonists of EP2 (AH-6809), EP4 (AH-23848), DP1 (BW-A868C), or DP2 (BAY-u3405) at 10 μM for 48 or 72 h. The cells were visualized by phase-contrast microscopy for the analysis of migratory and invasive abilities using Fibronectin-coated Transwell and Matrigel-coated Transwell plates, respectively. Scale bars: 100 μm. Data are from one experiment representative of three independent experiments with similar results.
